# Intelligent Tutoring Systems, Generative Artificial Intelligence (AI), and Healthcare Agents: A Proof of Concept and Dual-Layer Approach

**DOI:** 10.7759/cureus.69710

**Published:** 2024-09-19

**Authors:** Mohammed As'ad

**Affiliations:** 1 Emergency Department, Dr. Sulaiman Al-Habib Medical Group, Riyadh, SAU

**Keywords:** quality management in medical education and health care services, simulation in medical education, ai agents, generative ai, intelligent tutoring systems

## Abstract

This study introduces a novel methodology for enhancing intelligent tutoring systems (ITS) through the integration of generative artificial intelligence (GenAI) and specialized AI agents. We present a proof of concept (PoC) demo that implements a dual-layer GenAI validation approach that utilizes multiple large language models to ensure the reliability and pedagogical integrity of the AI-generated content. The system features role-specific AI agents, a GenAI-powered scoring mechanism, and an AI mentor that provides periodic guidance. This approach demonstrates capabilities in dynamic scenario generation and real-time adaptability while addressing key challenges in AI-driven education, such as personalization, scalability, and domain-specific knowledge integration. Although exemplified here through a case study in healthcare root cause analysis, the methodology is designed for broad applicability across various fields. Our findings suggest that this approach has significant potential for advancing adaptive learning and personalized instruction while raising important considerations regarding ethical AI application in education. This work provides a foundation for further research into the efficacy and impact of GenAI-enhanced ITS on learning outcomes and instructional design across diverse educational domains.

## Introduction

Educational technology has long sought to replicate the benefits of one-on-one human tutoring, with intelligent tutoring systems (ITS) emerging as a key tool in this pursuit. Since the 1970s, adaptive and interactive computer-based teaching systems have evolved, incorporating domain models, student models, teaching models, and user interfaces to provide tailored instruction [1,2]. While ITS have shown promise in various disciplines, including mathematics, physics, and computer programming, challenges persist in their adaptation and scalability and their handling of complex, open-ended domains [3-5].

Recent advancements in artificial intelligence (AI), particularly in machine learning and natural language processing, have significantly expanded ITS capabilities. The advent of Generative AI (GenAI) models, such as GPT-4, GPT-3.5, and Gemini, represents a paradigm shift in natural language processing and content generation [6]. These models offer unprecedented potential for generating dynamic, context-aware content, engaging in natural language interactions, providing instant feedback, and adapting explanations to individual learners. Concurrently, the development of AI agents has introduced new possibilities for creating engaging and interactive learning environments. These autonomous agents, which are capable of perceiving their environments and taking goal-oriented actions, enhance educational experiences by simulating real-world scenarios, providing personalized guidance, facilitating collaborative learning, and offering immediate feedback [7-9].

The integration of GenAI and AI agents within ITS frameworks marks the beginning of a new frontier in educational technology [10]. This integration promises to overcome longstanding challenges in ITS design, such as adaptability, scalability, and domain complexity. However, it also raises critical considerations regarding the reliability of AI-generated content, the potential perpetuation of biases, and the ethical implications of AI-driven personalization in education [11].

Despite the transformative potential of GenAI and AI technologies, several challenges impede their seamless integration into ITS. Particularly pressing challenges include ensuring the fidelity and precision of AI-generated content, engineering genuinely adaptive systems, developing scalable architectures applicable across diverse educational domains, harmonizing AI-driven personalization with structured pedagogy, and addressing ethical considerations inherent in AI-driven educational systems. The present study introduces a novel approach to addressing these challenges through a proof of concept (PoC) demonstration that uses GenAI and AI agents to implement ITS principles. We present a dual-layer AI validation methodology that enhances the reliability and pedagogical efficacy of AI-generated content and interactions. Our approach integrates multiple GenAI models within a unified ITS framework, thereby illustrating the application of core ITS principles using state-of-the-art AI technologies. We propose a flexible architecture that is adaptable to various educational domains and learning objectives, as illustrated through a case study in root cause analysis (RCA) training.

This research contributes to the field of AI in education by proposing an innovative dual-layer approach to AI validation, by exemplifying the integration of multiple GenAI models within a singular ITS framework, and by addressing critical challenges in personalization, adaptivity, and ethical considerations in AI-driven education. Through the presentation of a theoretical framework and demonstrated PoC implementation, this study aims to catalyze further research and development focused on the integration of advanced AI technologies and established ITS principles to offer a vision of a future steered by personalized, adaptive, and highly interactive AI-driven education.

Theoretical framework and literature review

ITS represent the convergence of cognitive science, educational psychology, and computer science and aim to deliver individualized instruction at scale. Rooted in Bloom's "2 sigma problem" [12], which demonstrated the efficacy of one-on-one tutoring, ITS design has been significantly influenced by cognitive architectures, such as Anderson's Adaptive Control of Thought-Rational (ACT-R) theory [13]. Anderson’s framework, which posits that cognitive skills develop through the transformation of declarative into procedural knowledge, has been instrumental in creating cognitive tutors who can decompose complex tasks into elemental skills. Nevertheless, ACT-R faces limitations when dealing with ill-structured problems - problems that are ambiguous, lack clear solutions, or involve complex and uncertain elements. This limitation has led to the exploration of alternative architectures, like State, Operator, and Result (SOAR) and Cognitive Learning Architecture for Robotics and Intelligent Optimization (CLARION), which offer promising avenues for managing complex, open-ended domains [14].

Adaptivity within ITS is intrinsically linked to Vygotsky's concept of the Zone of Proximal Development (ZPD), wherein systems dynamically modulate task difficulty based on the learner's current knowledge state [15]. This adaptivity is achieved through knowledge-tracing techniques that range from Bayesian approaches to cutting-edge deep learning methods [16]. The integration of metacognitive modeling and affect-aware tutoring represents a significant advancement. However, accurately capturing and responding to higher-order cognitive processes and emotional states remains an active area of research [17,18]. The emergence of Large Language Models (LLMs) and other GenAI technologies has brought about unprecedented advancements in educational technology. These models, based on transformer architectures [19], exhibit functionalities that profoundly resonate with integral ITS capabilities. Their prowess in natural language understanding and generation facilitates interactions that are more intuitive and contextually aware within educational environments [20]. The capability of models like GPT-4 to sustain multi-turn dialogs and preserve context over prolonged interactions portends the potential for more intricate tutoring dialogues [21].

A salient attribute of advanced LLMs is their capability for few-shot learning, which enables their adaptation to novel tasks with minimal explicit training [22]. This characteristic, by addressing the "knowledge engineering bottleneck" highlighted by Suraweera [23], potentially revolutionizes the updating and expansion of ITS knowledge bases. Recent strides in GenAI have also transcended textual data to encompass image generation and multimodal understanding and align with the cognitive theory of multimedia learning to offer the potential to craft enriched, multimodal learning experiences [24]. AI agents within educational frameworks can be envisaged as embodiments of intelligent tutoring principles that are capable of autonomous operations within learning environments [25]. Grounded in theories of embodied cognition [26] and situated learning [27], these agents can facilitate contextually relevant learning experiences and potentially augment learner engagement and comprehension. Drawing on social cognitive theory [28], pedagogical agents can enhance motivation, bolster self-efficacy, and promote deeper cognitive processing [29].

As AI systems become increasingly integral to educational frameworks, ensuring their reliability and validity is crucial. This necessity intersects with broader discussions on AI ethics and the development of trustworthy AI systems [30]. Explainable AI (XAI) methodologies are being rigorously explored to enhance the interpretability of AI-driven educational decisions in alignment with the pedagogical principles of constructive feedback and metacognitive development [31]. Robust bias detection and mitigation strategies are also indispensable for addressing both explicit and subtle biases that may manifest in language use or problem-solving methodologies [32]. As with dual-process theories in cognitive psychology [33], a dual-layer approach to AI validation in educational contexts could effectively mitigate the risks associated with AI-generated content. This strategy would involve an initial, rapid, intuitive evaluation followed by a more deliberate, analytical validation process and could potentially leverage multiple AI models with varying architectures or training paradigms.

The aim of integrating advanced AI into ITS is to achieve personalization levels that rival or surpass human tutoring [23]. This goal is supported by several theoretical frameworks, including Zimmerman's model of self-regulated learning [34] and Gardner's theory of multiple intelligences [35]. AI-driven systems could identify and cater to diverse aptitudes with greater flexibility than is possible using traditional instructional methods. Furthermore, Feuerstein's concept of dynamic assessment [36] aligns seamlessly with the capabilities of AI-driven adaptive systems. Recent research indicates that these systems can employ continuous assessment techniques to provide dynamic adjustments to educational content, thereby operationalizing dynamic assessment principles and enhancing learning efficiency across diverse educational needs [37].

The convergence of ITS principles with cutting-edge AI technologies presents unprecedented opportunities for advancing personalized, adaptive, and highly interactive educational experiences. However, this integration also necessitates rigorous investigation into the ethical implications, reliability, and pedagogical efficacy of these systems. As this field evolves, interdisciplinary collaboration will be crucial for realizing the full potential of AI-enhanced ITS while ensuring their alignment with fundamental educational objectives and ethical considerations.

## Technical report

Methodology and PoC implementation

This section delineates the methodological framework and provides a practical PoC demonstration of an ITS that uses GenAI and AI agents. The information provided here emphasizes the dual-layer approach employed for response generation and validation.

System Architecture

The ITS system architecture presented here is crafted to deliver a flexible, scalable, and adaptive learning experience. Its central components include a Theme and Scenario Database; a JSON file with predefined themes based on frequent medical incidents, each containing three detailed scenarios. When the user chooses a theme, the system randomly selects one scenario for the session. Multiple AI agents, each embodying distinct roles (e.g., Prescribing Physician, Pharmacist, Charge Nurse), are powered by GenAI models. This dual-layer AI approach generates and validates responses, thereby enhancing reliability and pedagogical efficacy. An AI mentor agent, using the dual-layer method, provides periodic guidance based on ongoing conversations. A Conversation Summarizer condenses extensive conversation histories to maintain context without overwhelming the AI models. The interactive chat interface facilitates learner engagement with AI agents without direct access to scenario details. The Domain Knowledge Base serves as a comprehensive repository of structured knowledge, learning objectives, and assessment criteria. The Student Model dynamically represents the student’s knowledge state, learning preferences, and progress. A Learning Analytics Module collects and analyzes data on student performance and system effectiveness for continuous enhancement.

The Dual-Layer AI Validation Approach

The dual-layer AI approach is integral to the system, as it encompasses both the AI agents and the mentor. The Initial Response Generation (Layer 1) employs an LLM, such as GPT-3.5-turbo, to produce an initial response based on the agent’s role, detailed scenario information, conversation history, and specific instructions. The Response Refinement and Validation (Layer 2) then implements another AI model, such as GPT-3.5-turbo, to undertake a second pass to refine and validate the initial response. This layer ensures several things, including adherence to the agent’s role and characteristics, compliance with predefined response rules, alignment with pedagogical objectives, and consistency with the previous responses and scenario details. It acts as a validator AI agent to guarantee that the response fulfills all necessary criteria. This dual-layer methodology substantially enhances the quality and dependability of the AI-generated responses by reducing inconsistencies and inappropriate content.

Implementation of ITS Principles

The system implements fundamental ITS principles through advanced mechanisms. Adaptive instruction, realized via the AI Agent Layer and Student Model, facilitates real-time adjustments based on learner responses and patterns. Cognitive modeling integrates traditional knowledge tracing with deep learning for knowledge state prediction. Metacognitive support generates reflective prompts, visualizes progress, and offers adaptive guidance on study strategies and self-regulation. Natural language processing interprets conceptual understanding from free-form responses to provide a detailed view of the learner’s cognitive state.

AI Agent Development

AI agents are crafted using a sophisticated combination of rule-based systems and machine-learning techniques. Each agent's behavior aligns with its professional role in medical incidents, and the agent maintains awareness of conversation history and scenario details. Response generation employs a dual-layer approach to ensure contextually appropriate and educationally supportive interactions. A coordination mechanism enables multiple agents to collaborate seamlessly to manage various tutoring aspects. Sentiment analysis allows agents to respond aptly to the learner’s emotional state to create a more empathetic learning environment.

Scenario Generation and Management

The system’s scenario generation capabilities provide diverse, pertinent, and adaptive learning experiences through several mechanisms. Utilizing GenAI, the system crafts varied problem scenarios, explanations, and examples. It dynamically adjusts the complexity of these scenarios based on the student's knowledge state and learning objectives. Additionally, the system generates scenarios that are contextually relevant to the learner's background, interests, and real-world applications of the subject matter. It also produces multimodal content, incorporating text, images, and potentially other media forms to address different learning preferences.

Mentor Agent

Through a dual-layer approach, the mentor agent acts as a guiding presence throughout the learning process. It intervenes after every five exchanges between the learner and other AI agents using the dual-layer method to generate and refine guidance. This ensures relevance and alignment with learning objectives while promoting in-depth analysis. Interventions, which are based on the accumulated conversation history, provide tailored prompts for reflection and further inquiry.

Performance Assessment and Feedback Systems

**Figure 1:**
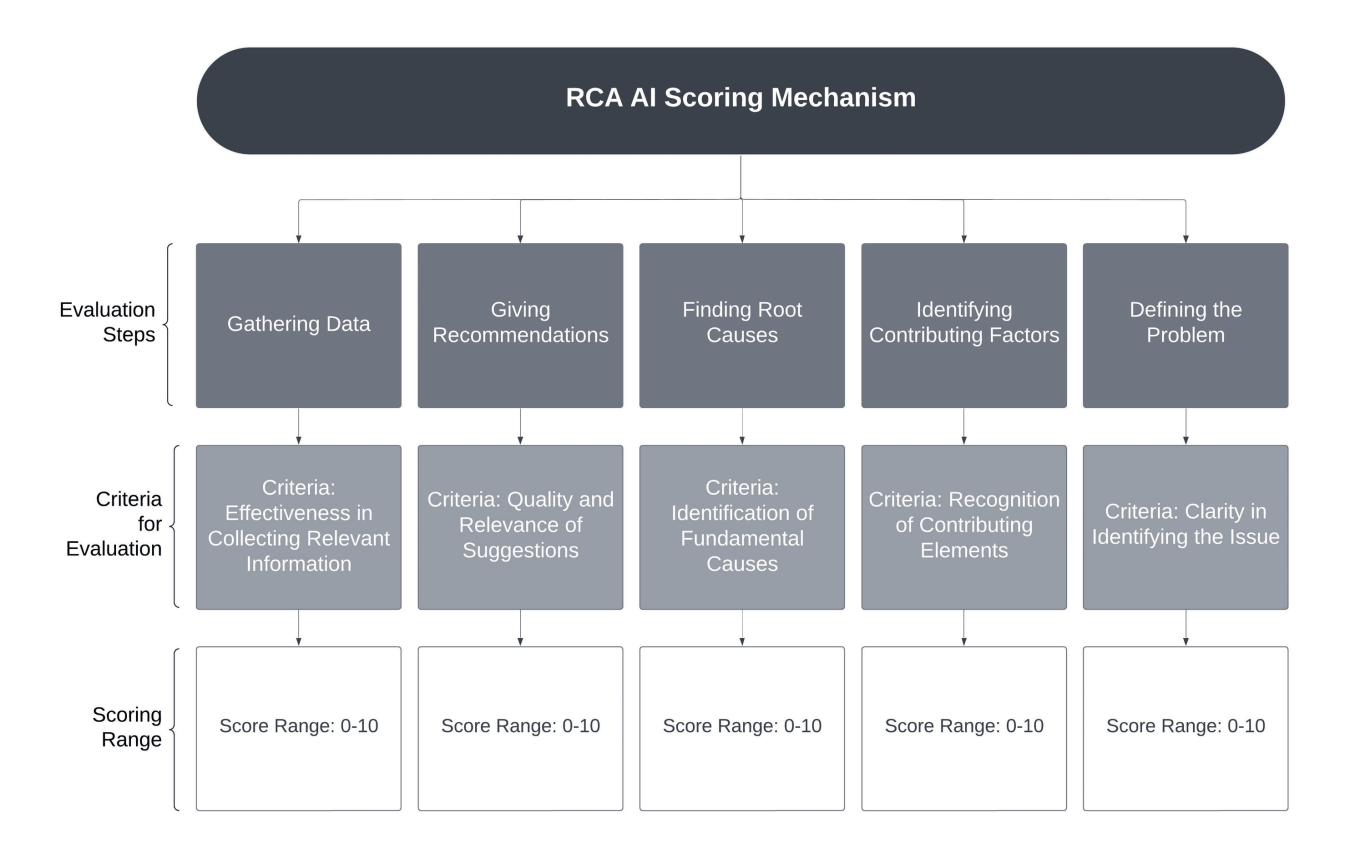
The scoring mechanism powered by generative AI to assess various aspects of learner interactions and progress. RCA: root cause analysis; AI: artificial intelligence Image created with Lucidchart (Lucid Software Inc., South Jordan, USA).

The performance assessment module evaluates the efficacy of the ITS in simulating real-world medical incident analysis and in delivering substantive feedback to learners. This module employs an algorithmic scoring mechanism powered by GenAI to assess various facets of learner interactions and progress (Figure 1). This structured scoring mechanism evaluates learner responses based on predefined criteria using a blend of rule-based and AI-driven techniques to ensure thorough and equitable assessment.

The core components include scenario detail extraction and AI-driven scoring. Scenario information extraction involves identifying key elements from the selected scenario, such as title, incident description, root causes, contributing factors, and recommendations. Learner feedback analysis divides and organizes feedback into manageable segments for detailed evaluation. AI-driven scoring operates by providing the AI with detailed instructions on assessing learner performance, covering problem definition, data gathering, identification of contributing factors, RCA, and recommendations. The AI uses a predefined template to score each feedback segment on a 0-10 scale, where 10 signifies perfect performance.

The feedback loop ensures continuous evaluation, progressively enhancing learner performance. Each step in the assessment process is scored on a scale of 0 to 10, with 10 indicating perfect performance and completion of all necessary aspects, while 0 indicates none of the required steps were addressed. The performance assessment module is implemented using JavaScript and OpenAI’s GPT model. A simplified version of this implementation can be found on GitHub [38]. This example illustrates the integration of AI-driven scoring within the ITS, illustrating the system’s ability to provide detailed, structured, and continuous feedback to learners. By utilizing advanced AI models, the performance assessment module ensures learners receive accurate and constructive evaluations, deepening their understanding of medical incident analysis and root cause investigation.

Conversation Management

The system integrates a summarization feature to manage extended conversations effectively. By periodically condensing the conversation history using a GenAI-powered summarizer, it preserves key points and the flow of dialogue while reducing the overall token count. This functionality enables the system to maintain context throughout prolonged learning sessions without exceeding model token limits, ensuring smooth and efficient handling of extended interactions.

User interaction flow

**Figure 2:**
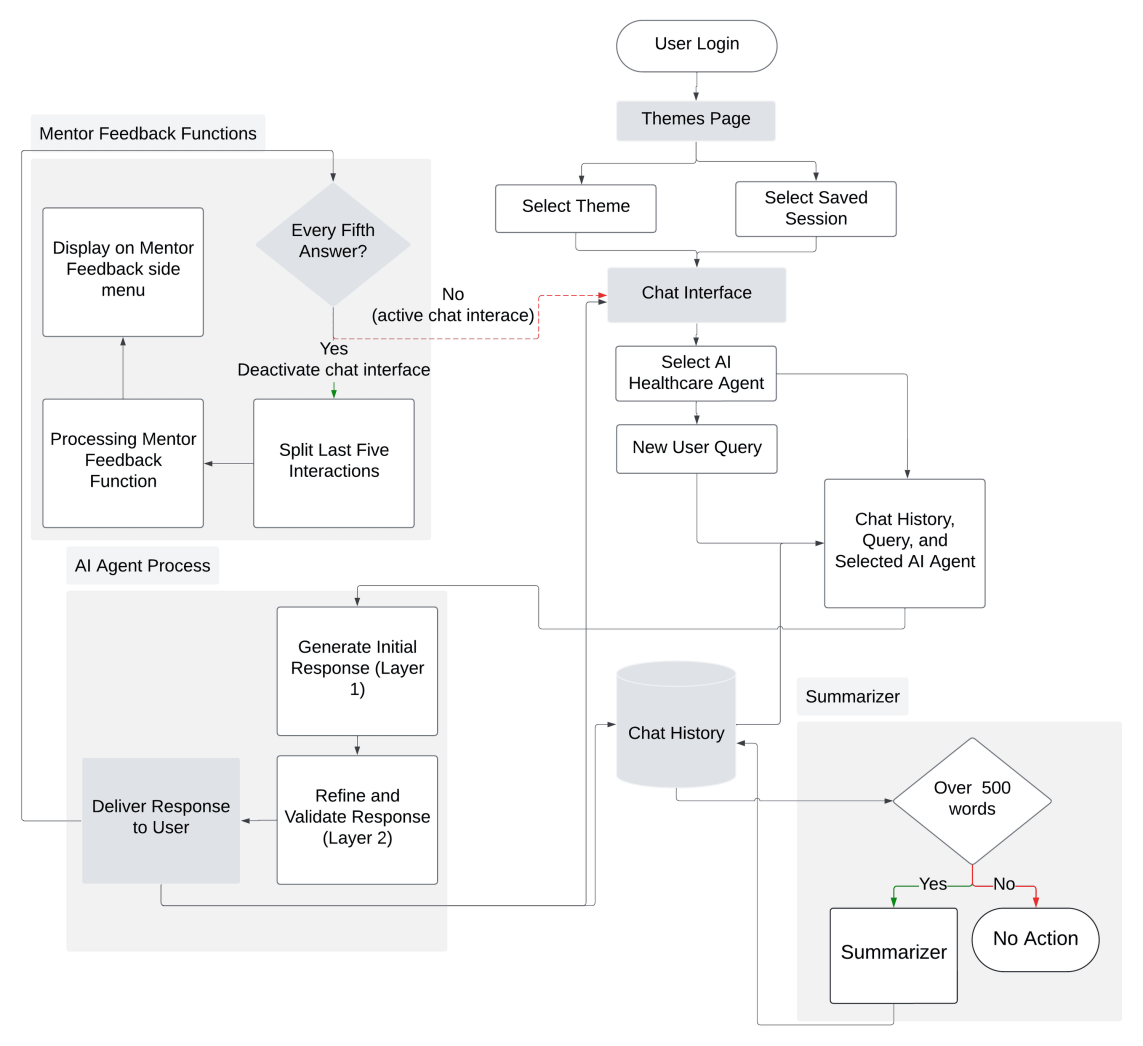
User and system interaction flows in the demo proof of concept. AI: artificial intelligence Image created with Lucidchart (Lucid Software Inc., South Jordan, USA).

The user interaction flow in the demo PoC of the ITS is meticulously designed to facilitate an efficient learning process (see Figure 2). It commences with user login, which provides access to the themes page. Users have the option of selecting a new theme or resuming a saved session, thereby ensuring both flexibility and continuity. Upon selecting a theme, users are directed to the chat interface, where they interact with various AI healthcare agents that simulate different professional roles within medical scenarios. When a query is submitted, the system processes the chat history, the query, and the selected AI agent to maintain contextual relevance. The ITS employs a dual-layer AI model. Initially, an AI agent generates a preliminary response using an LLM. This response is then refined and validated by a second AI model to ensure that the preliminary response has met the criteria for accuracy, relevance, and pedagogical effectiveness. A final response is then delivered to the user, completing the interaction cycle. Every fifth user interaction activates the mentor feedback function, which temporarily deactivates the chat interface to process the last five interactions. The system splits these interactions and generates mentor feedback, which is displayed on the mentor feedback side menu. This mechanism provides continuous guidance, aiding learners in reflecting on their progress and enhancing their problem-solving strategies. The system also includes a summarization feature for managing extended conversations. When the chat history exceeds 500 words, the summarizer condenses the conversation, preserving key points and logical flow while reducing the token count. This ensures that the system remains efficient throughout prolonged sessions. Overall, the interaction flow - from user login to mentor feedback and summarization - is crafted to offer a structured, responsive, and pedagogically sound learning environment.

**Figure 3:**
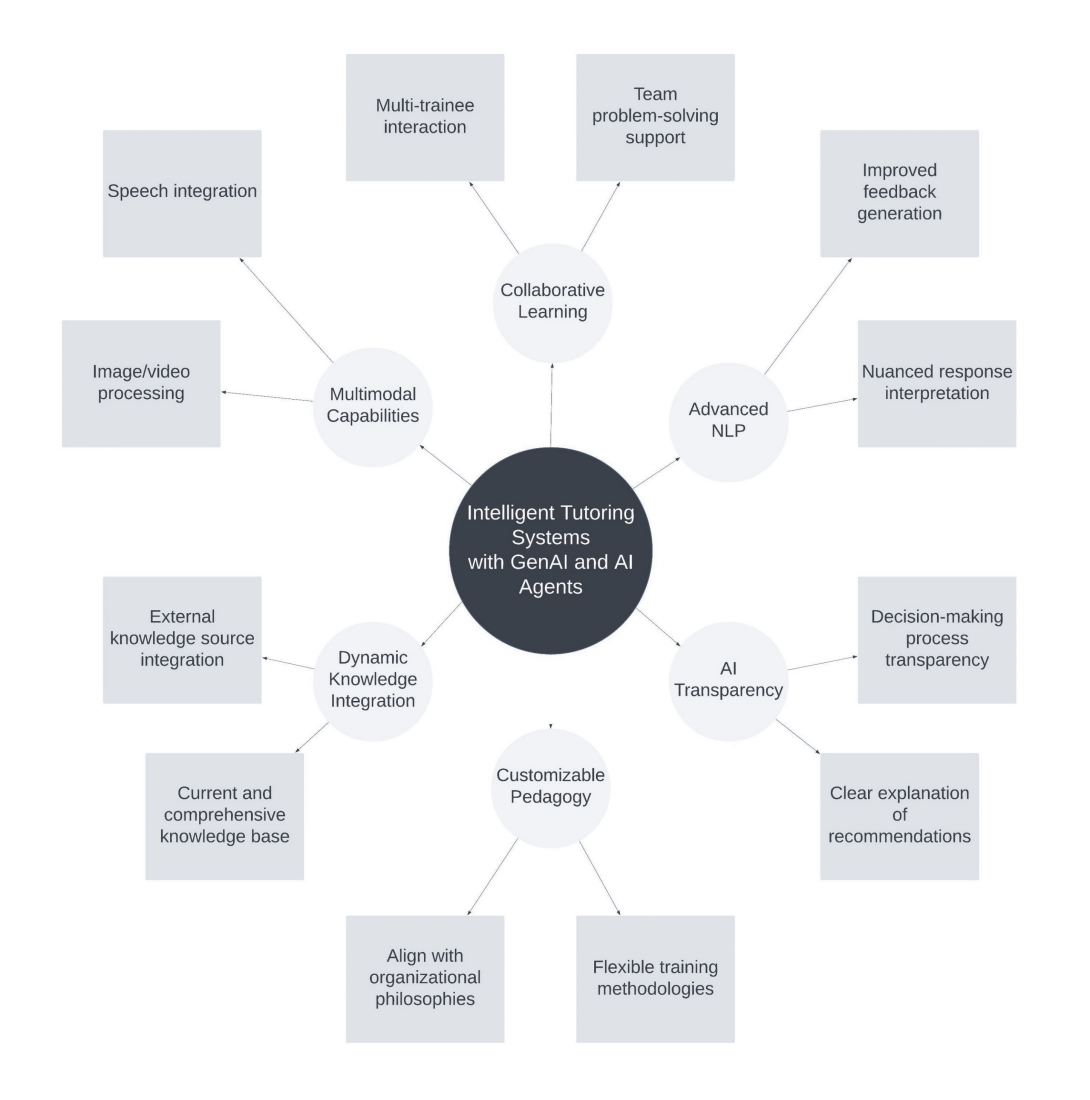
Framework for future research directions for intelligent tutoring systems with generative AI and AI agents. GenAI: generative artificial intelligence; NLP: natural language processing Image created with Lucidchart (Lucid Software Inc., South Jordan, USA).

In the next section, we will propose a comprehensive empirical evaluation strategy on the effectiveness and impact of our GenAI and AI agent-enriched approach (Figure 3).

## Discussion

Theoretical implications and potential impact

The implementation of the demo PoC using GenAI and AI agents represents a significant advancement in the field of AI-driven education. This section discovers the theoretical implications of our approach and its potential impact on various aspects of educational technology and practice.

Advancements in ITS Design

The demo PoC architecture and implementation methodologies suggest various ITS design innovations that potentially shape the sector. Tailored LLM integration with typical ITS components advances tutoring systems' flexibility and responsiveness. This hybrid approach aligns with the cognitive architecture theory proposed by Anderson et al. [13], particularly in its ability to model and respond to complex cognitive processes. The Demo PoC aims to tackle difficulties in ITS, namely by developing tutoring dialogues [39] that are more flexible and responsive to different situations. This is accomplished using the intrinsic characteristics of natural language processing in LLM.

A key innovation in this design is the dual-layer AI validation approach and multiple GenAI agents with different tasks. This can be a new model for ensuring the reliability and pedagogical soundness of AI-generated content in educational contexts. This method directly addresses one of the primary concerns in using GenAI for education - the potential for inaccuracies or inappropriate content [11]. By implementing a two-step process for generating and validating responses, the system enhances the quality and reliability of AI-generated content. However, it is important to note that this approach may introduce additional computational complexity and latency, potentially impacting real-time interactions.

The modular implementation of AI agents with specific roles demonstrates an advancement in ITS architecture. This design aligns with the component-based architecture proposed by Nkambou [40], allowing for more flexible and customizable systems that can be adapted to different educational domains and pedagogical approaches. Such modularity represents a significant step forward in addressing the scalability and domain-adaptability challenges often faced by traditional ITS. The system's ability to generate and adapt scenarios in real-time based on the learner's progress and preferences marks another important advancement in ITS design. This feature aligns with Vygotsky's [41] ZPD concept, enabling more precise support for learning experiences. The dynamic content generation capability represents a transformative change in how educational content is developed and delivered within ITS frameworks.

The incorporation of a mentor feedback system, where an AI mentor agent provides periodic guidance and feedback, represents a novel approach to ongoing learner support in ITS. This feature ensures that learners receive timely, relevant, and pedagogically sound guidance throughout their learning journey. It addresses the need for continuous, adaptive support in ITS. Another significant advancement is the implementation of a sophisticated scoring system that allows users to track their completion and progress throughout the session. By providing immediate and comprehensive feedback on learner performance, this feature addresses a critical aspect of effective tutoring. The use of both rule-based and AI-driven techniques in this scoring system represents a more nuanced and adaptive approach to learner evaluation in ITS design.

The inclusion of a conversation management feature that periodically condenses the conversation history addresses a common challenge in LLM-based systems: managing long-running conversations without losing context. This advancement enables the ITS to handle extended learning sessions more effectively, maintaining coherence and relevance throughout the tutoring process. Recent studies highlight the importance of context and chat history in enhancing the performance of LLMs, as these elements help maintain continuity and improve the accuracy of responses over time. Researchers have identified two main methods to enhance context handling in LLMs. The first method is external, summarizing and compressing the conversation into appropriate parts without losing important content [42]. The second method, as discussed by He et al. [43], involves developing LLMs that can handle larger contexts natively. This includes techniques such as attention approximation and hierarchical memory management. The Hierarchical Memory Transformer framework, for example, enables models to process long contexts more effectively by mimicking the hierarchical structure of human memory.

Personalization and Adaptivity

The demo PoC demonstrates significant advancements in personalization and adaptivity within ITS. The system's ability to generate and adapt scenarios in real-time based on the learner's progress and preferences points towards a future where learning materials are not static, but dynamically created to meet individual needs [10]. This is exemplified in our RCA training case study, where the system randomly selects one of three detailed scenarios associated with a chosen theme, providing a personalized starting point for each learning session. The implementation of multiple AI agents, each representing different roles in the medical incident (e.g., Prescribing Physician, Pharmacist, Charge Nurse), allows for a more nuanced and adaptive learning experience [25]. These agents maintain awareness of the full conversation history, scenario details, and their specific roles, enabling them to produce contextually appropriate responses that adapt to the learner's progress and inquiries.

Our PoC's mentor agent further enhances personalization by providing periodic interventions and guidance based on the ongoing conversation [44]. By intervening every five exchanges and basing its guidance on the accumulated conversation history, the mentor agent offers tailored prompts for reflection and suggestions for further inquiry, adapting to the learner's specific needs and progress. The scoring mechanism implemented in our PoC represents a significant advancement in adaptive assessment [45]. By using both rule-based and AI-driven techniques to evaluate learner responses, the system can provide personalized feedback and adapt to the difficulty of subsequent scenarios based on the learner's performance.

The conversation summarizer component of our system enables long-term adaptivity by condensing lengthy conversation histories while maintaining context. This allows the system to adapt to the learner's progress over extended learning sessions without losing relevant information or exceeding model token limits [20]. While these advancements in personalization and adaptivity show great promise, it is crucial to consider potential limitations. One concern is the potential creation of "echo chambers," a phenomenon where personalized content reinforces existing beliefs or knowledge, potentially limiting exposure to diverse perspectives [46]. In the context of adaptive learning systems, this could lead to reinforcing existing knowledge gaps if not carefully balanced with diverse learning experiences [47]. Future research should focus on striking the right balance between personalization and exposure to varied perspectives and challenges.

Scalability and Cross-Domain Application

The architecture of our demo PoC offers promising avenues for scalability and cross-domain application, extending beyond its current focus on RCA in healthcare. One key factor contributing to the system's scalability is its use of LLMs as the foundation for AI agents. LLMs have demonstrated remarkable generalization capabilities across various domains [48]. This characteristic suggests that our system could potentially adapt to new subject areas with minimal domain-specific engineering, a significant advantage over traditional ITS that often requires extensive customization for each new domain [3].

The modular nature of our AI agent implementation presents opportunities for cross-domain applications. By redefining agent roles and their associated knowledge bases, the system could be adapted to simulate various professional environments beyond healthcare. For instance, it could be reconfigured to model business negotiations, engineering design processes, or legal proceedings [10]. However, scaling to new domains is not without challenges. The effectiveness of the dual-layer AI validation approach in maintaining pedagogical integrity across diverse subjects requires further investigation. There's a risk that the generalization capabilities of LLMs might lead to a homogenization of teaching styles across different domains, potentially diminishing the rich diversity of pedagogical approaches that have evolved to suit specific subjects [47].

From a technical perspective, the computational demands of our current architecture, particularly the dual-layer validation process, may present challenges for large-scale deployment. As the system scales to accommodate more users and domains, optimizing resource usage becomes crucial. This might necessitate the exploration of more efficient AI architectures or distributed computing solutions [49]. Future research should focus on empirically validating the system's effectiveness across diverse domains, developing strategies to balance generalizability with domain-specific expertise, and addressing the technical challenges of scaling. These efforts will be crucial in realizing the full potential of our approach for creating adaptable, scalable, and effective AI-driven educational experiences across a wide range of subjects.

Methodological implications

The implementation of our demo PoC has significant methodological implications for research in educational technology and AI. Traditional educational assessment methods may not be sufficient to fully capture the effectiveness of AI-driven personalized learning experiences [44,45]. This opens up opportunities for research into new assessment methodologies that can account for the dynamic and personalized nature of AI-enhanced learning. Moreover, the integration of AI-generated content with human-created educational materials presents both challenges and opportunities for instructional design research [50]. Finding the right balance between AI-generated and human-created content and developing effective workflows for human educators to work alongside AI systems are areas ripe for further exploration.

The rapid pace of development in AI technology also necessitates new research methodologies capable of evaluating continuously evolving educational systems [51]. This presents an opportunity to develop new models for educational technology research that can keep pace with advancements in AI while maintaining rigorous empirical standards [52]. However, this rapid evolution also presents challenges in establishing stable, long-term studies to assess the true impact of these systems on learning outcomes [53]. There's a risk that the focus on technological advancement might overshadow the need for robust, longitudinal studies in educational research [47]. Additionally, the complexity of AI systems may create a "black box" effect, making it difficult for researchers to isolate and study specific variables influencing learning outcomes [49].

Recent advancements in GenAI and prompting methods further complicate these methodological considerations [54]. The emergence of adaptive prompting techniques and multimodal prompt engineering in educational contexts introduces new variables that researchers must account for [55,56]. For instance, AI systems that can adjust their responses based on a student's input style or interpret prompts containing text, images, and audio inputs may require novel assessment approaches [55,56]. Furthermore, the development of AI-assisted prompt generation and optimization tools, such as those being introduced by tech companies like Microsoft, could significantly alter how educators interact with AI systems [57]. These developments necessitate a reevaluation of instructional design research methodologies to effectively study the impact of evolving AI-human interactions in educational settings. Researchers must now consider how to measure the effectiveness of AI-augmented prompting techniques and their influence on learning outcomes [58], while also addressing ethical considerations in prompt engineering to ensure fairness and mitigate potential biases in AI-enhanced educational experiences [57].

Suggestions for next steps

The RCA AI case serves as a demonstration of the transformative potential for integrating GenAI and AI agents into ITS. This section outlines proposed empirical evaluations, potential system enhancements, and a long-term research agenda that builds upon the foundational work established herein.

Proposed Empirical Evaluation

To thoroughly evaluate the effectiveness and impact of our GenAI and AI agent-enhanced training approach, the paper proposes a comprehensive empirical evaluation strategy (Figure 3):

Phase 1: Comparative study: Researchers should initiate a randomized controlled trial with a diverse cohort of trainees. Participants would be assigned to one of three conditions: (1) The GenAI and AI agent-enhanced training system (RCA AI); (2) A conventional e-learning platform; (3) A control group using traditional training materials.

All groups would focus on the same subject matter (e.g., RCA) over a set period. Evaluation measures should include pre- and post-tests of domain knowledge, problem-solving skills assessments, and user experience surveys. Detailed interaction data from the RCA AI and e-learning platform groups should be collected to analyze learning patterns and system adaptivity.

Phase 2: Longitudinal field study: Following the initial study, researchers should conduct a prolonged field study in real organizational settings. This phase involves deploying the RCA AI system across multiple organizations over an extended duration (e.g., six months). The study aims to assess the system’s effectiveness in real-world conditions, adaptability to diverse training contexts, and long-term impact on skill development and application. Key factors to examine include trainee motivation, self-efficacy, and critical thinking skills development over time. Feedback from training managers on the system’s integration within existing training programs and its customizability across different domains and complexity levels would be invaluable.

Phase 3: Large-scale online deployment: The final phase should involve extensive deployment of the RCA AI system in online training environments. This approach would enable researchers to test the system’s scalability, evaluate its performance across diverse trainee populations, and gather substantial data for further refinement of the AI models. Comparative testing of various system components and configurations should be conducted to optimize the system based on real-world performance data. Researchers should also investigate the system’s effectiveness across multiple subject domains to gauge its generalizability and adaptability to varying complexity levels within organizational training contexts.

Potential Enhancements

The proposed enhancements for the AI-driven training system encompass a multifaceted approach to augment its capabilities and efficacy. Integrating multimodal input and output functionalities would significantly expand the system's versatility, enabling it to process and generate diverse content types, thereby creating a more immersive and personalized learning environment. This enhancement is intrinsically linked to the implementation of advanced natural language processing algorithms, which would refine the system's ability to interpret nuanced trainee responses and provide more precise, targeted feedback. The incorporation of collaborative learning features represents a pivotal development, facilitating team-based problem-solving and peer learning activities, which are particularly salient in organizational contexts that prioritize collective cognitive skills. Enhancing the transparency of AI decision-making processes is crucial for fostering trainee trust and promoting critical thinking skills, aligning with ethical AI principles and potentially yielding more effective learning outcomes. The dynamic integration of external knowledge sources emerges as a vital feature to maintain the system's relevance in rapidly evolving fields, ensuring a current and comprehensive knowledge base that adapts to emerging developments across various domains. Finally, the implementation of a framework for customizable pedagogical strategies would significantly enhance the system's flexibility, allowing for alignment with specific organizational training philosophies or methodologies, thus increasing its applicability across diverse training contexts. These proposed enhancements, collectively, aim to address the evolving requirements of AI-driven training systems, potentially offering more effective, adaptable, and engaging learning experiences across a spectrum of domains and complexity levels.

## Conclusions

This manuscript presents an AI-augmented training methodology that combines GenAI and AI agents to deliver adaptive, personalized learning experiences. At its core is a dual-layer GenAI validation framework: the first layer generates responses, while the second layer validates and refines these responses to enhance the reliability and effectiveness of AI-generated content. The integration of diverse GenAI models with specialized AI agents introduces a fundamental shift in ITS architecture, enabling dynamic scenario generation and real-time adaptability that surpasses traditional methods.

This approach addresses key challenges in AI-driven education, including personalization, scalability, and the integration of specialized domain knowledge. The system's advanced conversation management capabilities facilitate extended learning sessions with continuous, context-sensitive feedback. By outlining ethical considerations and empirical evaluation strategies, this study emphasizes the need for collaboration among educators, researchers, and technologists to ensure the development of impactful and ethically sound AI-driven learning experiences.

## References

[1] Corbett Corbett, AT AT, Koedinger KR, Anderson JR (1997). Intelligent Tutoring Systems. Handbook of Human-Computer Interaction. http://act-r.psy.cmu.edu/wordpress/wp-content/uploads/2012/12/173Chapter_37_Intelligent_Tutoring_Systems.pdf.

[2] Woolf BP (2010). Building Intelligent Interactive Tutors: Student-Centered Strategies for Revolutionizing e-Learning.

[3] Latham A (2022). Conversational intelligent tutoring systems: the state of the art. Women in Computational Intelligence: Key Advances and Perspectives on Emerging Topics.

[4] Lin CC, Huang AYQ, Lu OHT (2023). Artificial intelligence in intelligent tutoring systems toward sustainable education: a systematic review. Smart Learn Environ.

[5] VanLehn K (2011). The relative effectiveness of human tutoring, intelligent tutoring systems, and other tutoring systems. Educ Psychol.

[6] Imran M, Almusharraf N (2024). Google Gemini as a next generation AI educational tool: a review of emerging educational technology. Smart Learn Environ.

[7] Tan SC, Lee AVY, Lee M (2022). A systematic review of artificial intelligence techniques for collaborative learning over the past two decades. Comput Educ Artif Intell.

[8] Wongvorachan T, Lai KW, Bulut O, Tsai Y-S, Chen G (2022). Artificial intelligence: transforming the future of feedback in education. J Appl Test Technol.

[9] Xi Z, Chen W, Guo X (2023). The rise and potential of large language model based agents: a survey. arXiv preprint.

[10] Calo T, Maclellan C (2024). Towards educator-driven tutor authoring: generative AI approaches for creating intelligent tutor interfaces. L@S '24: Proceedings of the Eleventh ACM Conference on Learning @ Scale.

[11] Yu H, Guo Y (2023). Generative artificial intelligence empowers educational reform: current status, issues, and prospects. Front Educ.

[12] Bloom BS (1984). The 2 sigma problem: the search for methods of group instruction as effective as one-to-one tutoring. Educ Res.

[13] Anderson JR, Bothell D, Byrne MD, Douglass S, Lebiere C, Qin Y (2004). An integrated theory of the mind. Psychol Rev.

[14] Laird JE (2012). The Soar Cognitive Architecture. https://mitpress.mit.edu/9780262538534/the-soar-cognitive-architecture/.

[15] Raslan G (2024). The impact of the zone of proximal development concept (scaffolding) on the students problem solving skills and learning outcomes. BUiD Doctoral Research Conference.

[16] Piech C, Bassen J, Huang J, Ganguli S, Sahami M, Guibas LJ, Sohl-Dickstein J (2015). Deep knowledge tracing. Adv Neural Info Process Syst.

[17] Abdelshiheed M, Barnes T, Chi M (2023). How and when: the impact of metacognitive knowledge instruction and motivation on transfer across intelligent tutoring systems. Int J Artif Intell Educ.

[18] Abuazizeh M, Yordanova K, Kirste T (2021). Affect-aware conversational agent for intelligent tutoring of students in nursing subjects. Intelligent Tutoring Systems.

[19] Vaswani A, Shazeer N, Parmar N (2017). Advances in Neural Information Processing Systems 30: annual conference on Neural Information Processing Systems. Advances in Neural Information Processing Systems 30. I Guyon, U von Luxburg, S. Bengio, H. Wallach, R. Fergus, S. Vishwanathan, R. Garnett (eds): Neural Information Processing Systems Foundation.

[20] Huber SE, Kiili K, Nebel S, Ryan RM, Sailer M, Ninaus M (2024). Leveraging the potential of large language models in education through playful and game-based learning. Educ Psychol Rev.

[21] Lee D, Arnold M, Srivastava A (2024). The impact of generative AI on higher education learning and teaching: a study of educators’ perspectives. Comput Educ Artif Intell.

[22] Li M, Zhou H, Yang H, Zhang R (2024). RT: a Retrieving and Chain-of-Thought framework for few-shot medical named entity recognition. J Am Med Inform Assoc.

[23] Suraweera P (2007). Widening the knowledge acquisition bottleneck for intelligent tutoring systems. University of Canterbury.

[24] AlShaikh R, Al-Malki N, Almasre M (2024). The implementation of the cognitive theory of multimedia learning in the design and evaluation of an AI educational video assistant utilizing large language models. Heliyon.

[25] Grubaugh S, Levitt G, Deever D (2023). Harnessing AI to power constructivist learning: an evolution in educational methodologies. J Effect Teach Meth.

[26] Wilson M (2002). Six views of embodied cognition. Psychon Bull Rev.

[27] Lave J, Wenger E: Situated Learning (1991). Situated Learning: Legitimate Peripheral Participation.

[28] Bandura A (1986). Social Foundations of Thought and Action: A Social Cognitive Theory. Prentice Hall, Englewood Cliffs, NJ.

[29] Schroeder NL, Adesope OO, Gilbert RB (2013). How effective are pedagogical agents for learning? A meta-analytic review. J Educ Comput Res.

[30] Dignum V (2023). Responsible artificial intelligence - from principles to practice: a keynote at TheWebConf 2022. ACM SIGIR Forum.

[31] Gunning D, Aha D (2019). DARPA’s explainable artificial intelligence (XAI) program. AI Mag.

[32] Bender EM, Gebru T, McMillan-Major A, Shmitchell S (2021). On the dangers of stochastic parrots: can language models be too big?. FAccT '21: Proceedings of the 2021 ACM Conference on Fairness, Accountability, and Transparency.

[33] Evans JS (2008). Dual-processing accounts of reasoning, judgment, and social cognition. Annu Rev Psychol.

[34] Zimmerman BJ (2000). Chapter 2: attaining self-regulation: a social cognitive perspective. Handbook of Self-Regulation.

[35] Gardner HE (2011). Frames of Mind: The Theory of Multiple Intelligences. 35. Gardner HE: Frames of Mind: The Theory of Multiple Intelligences. Basic Books, New York; 2011..

[36] Feuerstein R, Rand Y, Hoffman MB (1981). The dynamic assessment of retarded performers: the learning potential assessment device, theory, instruments and techniques. Int J Rehabil Res.

[37] Laak K-J, Aru J (2024). AI and personalized learning: bridging the gap with modern educational goals. arXiv.

[38] (2024). Scoring agent implementation. https://github.com/masad08/misc/blob/main/scoring.js.

[39] Katz S, Albacete P, Chounta I-A, Jordan P, McLaren BM, Zapata-Rivera D (2021). Linking dialogue with student modelling to create an adaptive tutoring system for conceptual physics. Int J Artif Intell Educ.

[40] Nkambou R (2010). Modeling the domain: an introduction to the expert module. Advances in Intelligent Tutoring Systems.

[41] Vygotsky LS, Cole M (1978). Mind in Society: Development of Higher Psychological Processes.

[42] Fei W, Niu X, Zhou P, Hou L, Bai B, Deng L, Han W (2023). Extending context window of large language models via semantic compression. arXiv.

[43] He Z, Qin Z, Prakriya N, Sun Y, Cong J (2024240506067). HMT: Hierarchical memory transformer for long context language processing. arXiv.

[44] González-Calatayud V, Prendes-Espinosa P, Roig-Vila R (2021). Artificial intelligence for student assessment: a systematic review. Appl Sci.

[45] Ouyang F, Dinh TA, Xu W (2023). A systematic review of AI-driven educational assessment in STEM education. J Stem Educ Res.

[46] Cinelli M, De Francisci Morales G, Galeazzi A, Quattrociocchi W, Starnini M (2021). The echo chamber effect on social media. Proc Natl Acad Sci U S A.

[47] Popenici SA, Kerr S (2017). Exploring the impact of artificial intelligence on teaching and learning in higher education. Res Pract Technol Enhanc Learn.

[48] Brown T, Mann B, Ryder N (2020). Language models are few-shot learners. Adv Neur Infor Proc Syst.

[49] Hassija V, Chamola V, Mahapatra A (2024). Interpreting black-box models: a review on explainable artificial intelligence. Cogn Comput.

[50] Michel-Villarreal R, Vilalta-Perdomo E, Salinas-Navarro DE, Thierry-Aguilera R, Gerardou FS (2023). Challenges and opportunities of generative AI for higher education as explained by ChatGPT. Educ Sci.

[51] Guo S, Zheng Y, Zhai X (2024). Artificial intelligence in education research during 2013-2023: a review based on bibliometric analysis. Educ Inf Technol.

[52] Cain W (2024). Prompting change: exploring prompt engineering in large language model AI and its potential to transform education. TechTrends.

[53] Aylward RC, Cronjé JC (2022). Paradigms extended: how to integrate behaviorism, constructivism, knowledge domain, and learner mastery in instructional design. Educ Technol Res Devel.

[54] Almasri F (2024). Exploring the impact of artificial intelligence in teaching and learning of science: a systematic review of empirical research. Res Sci Educ.

[55] Bengesi S, El-Sayed H, Sarker MK, Houkpati Y, Irungu J, Oladunni T (2023). Advancements in generative AI: a comprehensive review of GANs, GPT, autoencoders, diffusion model, and transformers. IEEE Access.

[56] Barbosa PLS, Carmo RAF, Gomes JPP, Viana W (2024). Adaptive learning in computer science education: q scoping review. Educ Inform Technol.

[57] Wan X, Sun R, Nakhost H, Dai H, Eisenschlos JM, Arik SO, Pfister T (2023). Universal self-adaptive prompting. arXiv.

[58] Walter Y (2024). Embracing the future of artificial intelligence in the classroom: the relevance of AI literacy, prompt engineering, and critical thinking in modern education. Int J Educ Technol Higher Educ.

